# Establishment and primary clinical application of competitive inhibition for measurement of augmenter of liver regeneration

**DOI:** 10.3892/etm.2013.1140

**Published:** 2013-06-04

**Authors:** NA WANG, HANG SUN, LIN TANG, JIANCHUAN DENG, YA LUO, HUI GUO, QI LIU

**Affiliations:** Institute for Viral Hepatitis and Department of Infectious Diseases, Key Laboratory of Molecular Biology for Infectious Diseases, Ministry of Education, The Second Affiliated Hospital, Chongqing Medical University, Chongqing 400010, P.R. China

**Keywords:** human serum augmenter of liver regeneration, protein purification, monoclonal antibody, competitive inhibition

## Abstract

The aim of the present study was to establish a quantitative method for the measurement of serum human augmenter of liver regeneration (hALR) using competitive inhibition that is applicable in the clinic. A monoclonal antibody to hALR was used as the primary antibody and the pure hALR protein was used as a standard for competition with Eu^3+^-labeled hALR (Eu^3+^-hALR) to plot a standard curve. Serum samples from 90 patients with various liver diseases due to hepatitis B virus (HBV) infection were used for a competitive reaction with Eu^3+^-hALR. A regression analysis of the results was performed using the standard curve to calculate the serum concentration of hALR. The minimum detectable value using direct competitive measurement established by Eu^3+^-hALR was 1 ng/ml, with a positive linear correlation within the range of 200 ng/ml. In the sera of the 90 patients, the hALR level in the severe hepatitis group was the highest, followed by that in the acute hepatitis group. The serum hALR levels in the cirrhosis and chronic hepatitis groups were significantly higher compared with those in the normal control groups (P<0.01). The direct competitive measurement method of serum hALR established in the present study has high sensitivity, specificity, stability and reliability, meets clinical requirements and may be used as potential index in clinical tests.

## Introduction

Augmenter of liver regeneration (ALR) is a non-specific hepatocyte growth-promoting factor with heat stability, identified by Hagiya *et al*(1) in 1994 during a study of hepatic stimulator substance (HSS), and is different from hepatocyte growth factor (HGF) (2). Similar to insulin-like growth factor (IGF) and epidermal growth factor (EGF), ALR plays an important role in the regeneration of hepatocytes (3). In order to further understand the human ALR (hALR) concentration of serum and study the association of hALR with various liver diseases, particularly with the different stages of type-B hepatitis, it is necessary to investigate the serum hALR concentration in various types and extents of hepatitis and cirrhosis, and the correlation of hALR and disease in detail. According to the classical immunology theory (4), we aimed to establish a reliable method for measuring hALR using a directly competitive inhibition reaction, which is different from previous double antibody sandwich or indirect enzyme-linked immunosorbent assays (ELISAs). Eu^3+^-labeled hALR was used to compete with the pure hALR protein as an antigen in the competitive inhibition, and an anti-hALR monoclonal hybridoma cell line was used to produce anti-hALR monoclonal antibody; these processes established a direct competitive method for measuring serum hALR to aid the understanding of serum hALR concentration and its significance in various liver diseases.

## Materials and methods

### Materials and reagents

Recombinant plasmid pQE30-hALR was constructed in the Institute for Viral Hepatitis (Chongqing Medical University, Chongqing, China), as previously described (5). The polyhistidine protein purification kit (Ni^2+^-NTA Resin) was purchased from Qiagen GmbH (Hilden, Germany) and the capillary electrophoresis (CE) system (PACE 5500) was purchased from Beckman (Beckman Coulter Inc., Brea, CA, USA). BALB/c mice were provided by the Experimental Animal Centre of Chongqing Medical University (Chongqing, China). Eu^3+^ chelator, Eu^3+^-labeled dilution solution, fluorescence enhancer, time-resolved fluorometer, automicroplate washer and plate shaker were purchased from Sym-Bio Life Science (Zhejiang, China).

### Serum samples

The serum samples were collected from patients in the outpatient or inpatient ward of the Department of Infectious Diseases, the Second Affiliated Hospital, Chongqing Medical University between September 2005 and April 2006. The diagnosis met with the Viral Hepatitis Prevention & Treatment Strategy released by the Chinese Society of Hepatology and Society of Infectious Diseases (2005)(6). The blood samples were collected in the morning after fasting, incubated at 37˚C for 2 h, centrifuged at 1,200 × g for 10 min and stored at −20°C. This study complied with the Declaration of Helsinki, and was approved by the Ethics Committee of the Second Affiliated Hospital of Chongqing Medical University. All participants provided written informed consent.

## Methods

### Preparation of antigens and antigen labeling

i) Pronucleus expression of hALR: following identification, the recombinant plasmid pQE30-hALR was transformed into *E. Coli* SG13009 for expression induced by IPTG. The product was identified with 15% SDS-PAGE. ii) Affinity chromatography purification of hALR: hALR protein was purified using Ni-NTA and identified using 15% SDS-PAGE and capillary electrophoresis. iii) hALR labeling: purified hALR protein was labeled with Eu3+ via DTTA chelation (7) and the labeled product was identified by 15% SDS-PAGE and time-resolved fluorescence (TRF) immunoassay (TRFIA).

### Antibody preparation and identification

Anti-hALR hybridoma (AAMA) cells were established in our laboratory (8) and were cultured and inoculated intraperitoneally into BALB/c mice (9) as previously described. SP2/0 myeloma cells were used as a negative control. The harvested ascitic fluid was tested for the reactivity of hALR proteins and human albumin using an ELISA and immunoblot assay.

### Establishment of the measuring method and clinical application

Direct antigen competition was used to coat the 96-well ELISA plate with an optimal working concentration of anti-hALR (monoclonal antibody 100 μl/well, 1:800) at 4°C overnight. BSA/PBST was added (3%, 200 μl/well) and the well was blocked at 37°C for 3 h. Standard purified hALR protein (50 μl, 2 μg/ml) diluted with PBS at 1:10, 1:50, 1:100, 1:500, 1:1,000 and 1:5,000 was mixed with 50 μl Eu^3+^-hALR in the same reaction well for competitive inhibition reaction in the plate shaker and incubated at 37°C for 1 h. Three parallel wells were used for each concentration. Fluorescence enhancing solution (100 μl) was added to each well, the plates were incubated at 37°C for 5 min and the resulting samples were analyzed by TRFIA to create a standard curve. The serum samples from the patients with various liver diseases were used to replace the standard protein and competitively react with Eu^3+^-hALR in the blank, negative antibody control and negative quality control serum wells. A regression analysis of the results was performed using the standard curve to calculate the hALR concentration in the sera of the 90 patients with various liver diseases. Calf serum was used as a medium to prepare serial concentrations of hALR at 5, 10, 20 and 40 ng/ml to be detected with the direct competitive assay, and then the coefficient of recovery and the variation coefficient were calculated.

### Statistical analysis

The data are expressed as mean ± SD. SPSS 13.0 (SPSS, Inc., Chicago, IL, USA) was used to compare intra-group differences using a Student’s t-test. P<0.05 was considered to indicate a statistically significant result.

## Results

### Antigens and antigen labeling

**Figure 1:**
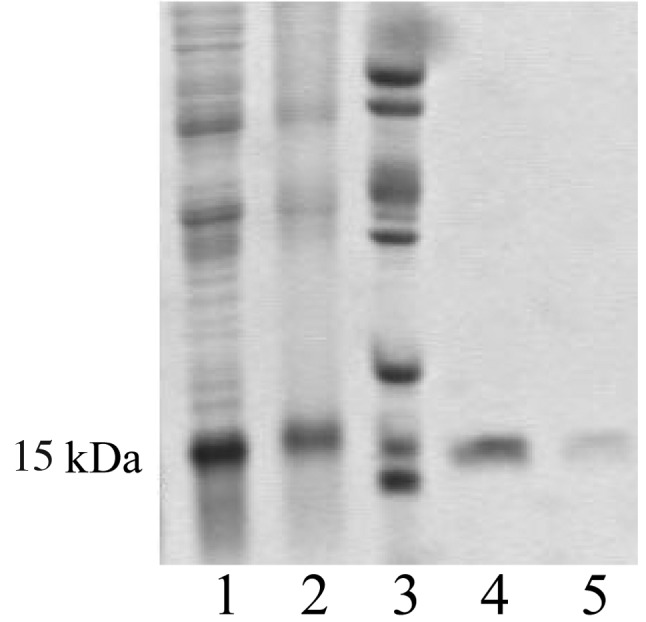
Purification of human augmenter of liver regeneration (hALR). Lanes 1 and 2: induction of SG(pQE30-hALR); lane 3: protein marker; lanes 4 and 5: purification of SG(pQE30-hALR) 15 and 5 mg/ml, respectively.

**Figure 2:**
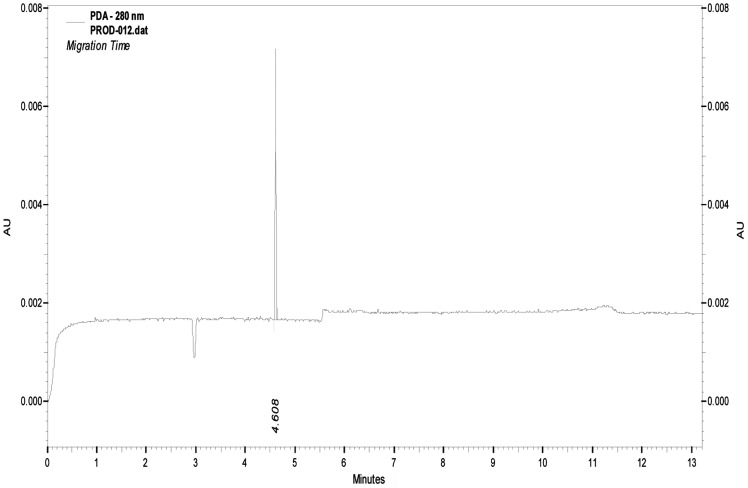
Identification of purified human ugmenter of liver regeneration (hALR) by capillary electrophoresis (CE).

The recombinant plasmid pQE30-hALR showed high expression in the host bacteria and the molecular weight of the product was approximately 15 kDa (Fig. 1). Following purification with affinity chromatography, the protein was identified as one band by SDS-PAGE with a purity of 90% by CE (Fig. 2).

### Antibody preparation and identification

**Figure 3:**
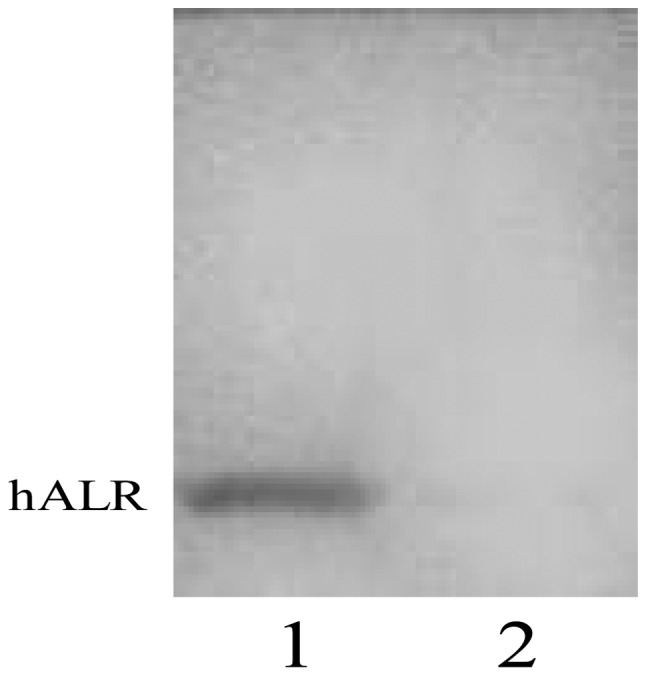
Reactivity of anti-hALR monoclonal antibody. Lane 1: hALR expressed in SG13009; lane 2: human serum albumin. hALR, human augmenter of liver regeneration.

The harvested anti-hALR monoclonal antibody ascites were measured by ELISA with an optimal working concentration of 1:800 (Table I). Western blotting showed the monoclonal antibody of hALR protein as a single band without interaction with the natural albumin protein in human serum (Fig. 3).

### Establishment and application of serum measurement

**Figure 4:**
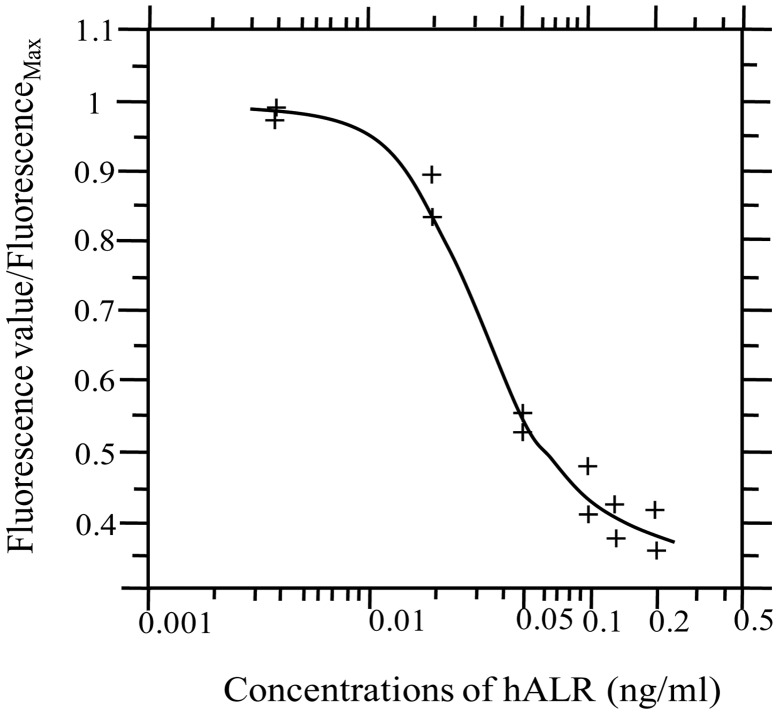
The standard curve of human augmenter of liver regeneration (hALR).

A standard curve for competitive inhibition of Eu^3+^-hALR and hALR was constructed. The fluorescence values of standard hALR and Eu^3+^-ALR at various concentrations were measured by TRF. A standard curve was constructed using matched software (Sym-Bio Life Science, Zhejiang, China), with the concentration of standard hALR on the x-axis and the ratio of fluorescence value in reaction wells/measured maximal fluorescence value on the y-axis, as shown in Fig. 4.

### Serum sample measurement

As shown in Table II, the concentrations of hALR in the sera of patients with various liver diseases (acute hepatitis, chronic hepatitis, cirrhosis and severe hepatitis) were significantly higher compared with that in the normal control group (P<0.01).

### Recovery percentage and variation coefficient

Using calf serum as a medium, the coefficient of recovery was detected. Table III shows the coefficient of recovery at four concentration levels and the variation coefficient obtained from the repeats.

## Discussion

ALR is a recently discovered growth factor that promotes liver regeneration and, similar to other cellular factors such as IGF and EGF, plays an important role in the regeneration of hepatocytes. We aim to understand the hALR concentration in the sera of patients with hepatitis and cirrhosis, and further investigate its interaction with these diseases.

In previous studies (10,11), serum hALR has been measured using a double antibody sandwich or indirect ELISA method. However, classical immunology (4) clearly suggests that a double antibody sandwich ELISA is not able to provide simultaneously two binding sites for two antibodies on small molecular antigens. In addition, large proteins in the serum may affect the binding of small molecular antigens to antibodies in an indirect ELISA. Therefore, the previously reported methods are not appropriate. In the present study, we established a method for measuring serum hALR in liver diseases of varying severity using competitive inhibition against small molecular antigens. Direct competition is simple and fast, and requires only one rinse and two additions of samples. Therefore, we selected direct antigen competitive inhibition as the response model. Through the competitive combination between Eu^3+^-hALR and hALR in serum with the coated monoclonal Abs, we established a measurement method for serum hALR.

The results indicated that the serum level of hALR in patients with viral hepatitis was higher than that in normal serum. In particular, severe hepatitis had the highest hALR level, which was 15-fold that of normal serum and 6 to 7-fold that of common hepatitis; the hALR level in acute hepatitis was 3-fold that of normal serum, and cirrhosis and chronic hepatitis had 3-fold higher serum hALR levels than normal serum. The hALR levels determined in the present study were higher than those reported in a previous study (10,11). This may be due to the different methodologies used. The double antibody sandwich or indirect ELISA used previously has insufficient sensitivity for small molecular antigens, is not able to provide two binding sites for two antibodies and is affected by temporal resistance which results in antigens being partially undetected and so is not able to reflect the actual levels of hALR in samples. We used a competitive inhibition model to avoid these challenges and provide a higher detection rate.

We noted that the serum hALR levels increased quickly in severe hepatitis caused by hepatitis B virus (HBV) infection. The reason for this increase is due to hepatocytes undergoing marked necrosis and releasing large amounts of hALR into the blood accompanied by increased secretion from other organs, including the kidney (12) and pancreas (13), and extensive damage to hepatocytes decreasing the binding and metabolism of hALR, resulting in a higher detection rate. The compensatory increase of secretion is closely correlated with liver regeneration and may be considered as an acute reaction of the body to liver damage. The cause of the increase of serum hALR in acute B-type hepatitis is similar to that in severe hepatitis, i.e., increased release, reduced binding and reduced metabolism of hepatocytes due to damage. In the present study, the chronic B-type hepatitic and B-type cirrhosis patients were patients in the inpatient ward, who had clear symptoms of chronic active hepatitis or decompensated cirrhosis, damaged liver function and hepatocyte damage of various extents. Therefore, the serum detection rate was high. In summary, the serum hALR concentration in various severities of liver disease is closely correlated with the extent of hepatocyte damage while whether hALR has a pro-regenerative effect on the hepatocytes depends upon the condition of the hepatocytes.

Based on the present results, the competitive inhibition method has sufficient accuracy, specificity and sensitivity to meet the requirements of clinical application. Through continuous optimization and widespread practice, this method may be used as a clinical diagnostic index for liver diseases and other diseases associated with hALR (such as urinary system diseases) (14), and to provide new theoretical evidence and a measurement method for the diagnosis and differential diagnosis of associated diseases.

## Figures and Tables

**Table I:** OD_450_ of hALR combined with mouse ascites in different concentrations.

	Concentration of mouse ascites diluted with PBS (v/v)				
	
Mouse ascites	1:200	1:400	1:800	1:1000	1:2000
SP2/0 ascites	0.214±0.08	0.184±0.05	0.155±0.05	0.164±0.08	0.138±0.06
AAMA ascites	0.985±0.15^a^	0.939±0.07^a^	0.896±0.04^a^^b^	0.785±0.01^a^	0.671±0.06^a^

Compared with corresponding concentration of SP2/0 ascites,

a P/N>2.1,

b P/N maximum value.

ELISA, enzyme-linked immunosorbent assay.

**Table II:** Serum hALR levels in 90 patients with various liver diseases.

Clinical group	No. of cases	hALR concentration (ng/ml)
Normal control	10	3.77±1.55
Acute hepatitis	5	10.14±3.26^a^
Chronic hepatitis	30	8.44±2.78^a^
Cirrhosis	30	10.11±4.32^a^
Severe hepatitis	15	57.34±18.96^a^

a P<0.01 vs normal control.

hALR, human augmenter of liver regeneration.

**Table III:** Recovery percentages of the direct competitive reaction.

Addition hALR (ng/ml)	Repeated wells (n)	Detected concentration hALR (ng/ml)	Recovery percentage (%)	Variation coefficient (%)
5.0	3	4.16	83.2	7.12
10.0	3	8.52	85.2	4.84
20.0	3	18.73	93.7	7.35
40.0	3	33.54	83.9	9.86
